# Medicaid payment rates and nursing home staffing levels across ownership types

**DOI:** 10.1093/geroni/igag087

**Published:** 2026-07-22

**Authors:** Edward Alan Miller, Elizabeth Simpson, Marc A Cohen, John R Bowblis

**Affiliations:** Department of Gerontology, Donna M. and Robert J. Manning College of Nursing & Health Sciences, University of Massachusetts Boston, Boston, Massachusetts, United States; LeadingAge Center for Long-Term Services & Supports @ UMass Boston, Gerontology Institute, Donna M. and Robert J. Manning College of Nursing and Health Sciences University of Massachusetts Boston, Boston, Massachusetts, United States; Department of Gerontology, Donna M. and Robert J. Manning College of Nursing & Health Sciences, University of Massachusetts Boston, Boston, Massachusetts, United States; LeadingAge Center for Long-Term Services & Supports @ UMass Boston, Gerontology Institute, Donna M. and Robert J. Manning College of Nursing and Health Sciences University of Massachusetts Boston, Boston, Massachusetts, United States; Department of Gerontology, Donna M. and Robert J. Manning College of Nursing & Health Sciences, University of Massachusetts Boston, Boston, Massachusetts, United States; LeadingAge Center for Long-Term Services & Supports @ UMass Boston, Gerontology Institute, Donna M. and Robert J. Manning College of Nursing and Health Sciences University of Massachusetts Boston, Boston, Massachusetts, United States; Department of Economics, Farmer School of Business, Miami University, Oxford, Ohio, United States; Scripps Gerontology Center, Miami University, Oxford, Ohio, United States

**Keywords:** Medicaid reimbursement, Licensed nurses, Nurse aides, Nursing home quality

## Abstract

**Background and Objectives:**

Medicaid is the largest payer for long-stay nursing home care, yet the relationship between payment and quality is understudied. Given the relationship of staffing to quality of care, this study aimed to investigate the association between Medicaid payment rates and nursing staff levels in nursing homes, both overall and by ownership type (for-profit, not-for-profit, government).

**Research Design and Methods:**

The study employed a retrospective, cross-sectional analysis using a nationwide, facility-level database, comprising facility-level Medicaid payment rates collected directly from the states, staffing levels derived from the Payroll-Based Journal (PBJ), and facility and resident characteristics from the Medicare Cost Reports and Certification and Survey Provider Enhanced Reporting (CASPER). Multivariate regression with state fixed effects was used to assess the relationship between Medicaid payment and staffing levels. The final sample accounted for 9,510 freestanding nursing homes across the United States.

**Results:**

Results demonstrate significant associations between Medicaid payment rates and total, nurse aide, and registered nurse staffing levels. Medicaid payment rates were not consistently associated with licensed practical nurse staffing levels. Findings did not vary across not-for-profit, for-profit, and government nursing homes.

**Discussion and Implications:**

Medicaid reimbursement levels affect nursing staff levels in nursing homes, regardless of ownership type. Policymakers should consider Medicaid payment rates when setting nursing home policy to support staffing and sustain quality of care.

Innovation and Translational Significance: This study examines national, facility-level Medicaid payment rates and their association with nursing staff levels and explores how this association varies across not-for-profit, for-profit, and government-owned nursing homes. Our analysis identified that higher Medicaid payment rates were associated with higher staffing levels across all ownership types for total, registered nurses, and nurse aides. This highlights the key role that state Medicaid payment policies play in shaping resident well-being, given the importance of staffing for quality. Policymakers and advocates seeking to improve resident quality of care should consider this association when advocating for policy change at the federal and state levels.

Nursing homes (NHs) provide both short-term rehabilitative and long-term care to older adults, with nursing staff providing most of this direct care. Nursing staff consists of nurse aides and two types of licensed nurse staff, registered nurses (RNs) and licensed practical nurses (LPNs). Nursing staff is an important factor contributing to the overall quality of care in NHs, though the documented effect has been inconsistent across studies due largely to variation in how staffing and quality outcomes are measured (Armijo-Olivo et al., 2020; Backhaus et al., 2014; Clemens et al., 2021; Millar et al., 2024). Nevertheless, multiple studies document an association between higher nursing staff levels and fewer deficiency citations (Bowblis & Roberts, 2020; Clemens et al., 2021; Harrington et al., 2000; Millar et al., 2024), consumer complaints to regulators (Peterson et al., 2022), lower rates of hospitalization (Clemens et al., 2021; Grabowski et al., 2008), and better resident clinical conditions, such as pressure injuries and the use of antipsychotic medications (Clemens et al., 2021; Millar et al., 2024).

## Nursing staff levels in nursing homes

The Centers for Medicare and Medicaid Services (CMS) does not provide a mathematical formula, nor is there a standard industry process, for determining the appropriate level of nursing staff for a particular facility; instead, staffing levels are determined by a combination of regulatory, structural, and economic factors. Federal regulations require only that NHs have both licensed nurses and nurse aides available 24 hr a day and that an RN must be present for at least 8 hr of every day (42 C.F.R. §483.35). As a result, total nursing staff levels vary considerably nationwide, averaging 3.76 hr per resident day (HPRD) in 2022, but with the lowest 10% of NHs having less than 2.79 HPRD and the highest 10% having greater than 4.88 HPRD (White et al., 2023). Several states have staffing minimums in place, many of which were implemented or strengthened in the wake of the COVID-19 pandemic (Musumeci et al., 2022). These minimum staffing standards range from nonexistent to as high as 3.6 HPRD in Florida (MACPAC, 2022). However, outside of minimum staffing standards, multiple factors could impact nursing staff levels, including resident acuity, local practice standards, workforce availability, and organizational practices that affect the recruitment and retention of nursing staff (Harrington et al., 2007; Kennedy et al., 2020).

## Nursing home ownership

A structural aspect that receives significant attention from NH stakeholders and which may be especially relevant to nursing staff levels is NH ownership. For-profit and not-for-profit NHs are thought to have different financial incentives, which could drive operational decision-making (Huang & Bowblis, 2018; Weech-Maldonado et al., 2012). For-profit NHs can allocate revenues without restrictions on profit distribution, for example, returning earnings to owners or shareholders. By contrast, not-for-profit NHs are subject to a non-distribution constraint which prohibits them from distributing profits (Hansmann, 1980). This non-distribution constraint may encourage greater reinvestment of revenues into the organizational mission, including staffing, resident care, and quality improvements. Additionally, not-for-profits do not pay certain taxes and may receive donations that provide a tax advantage to the donor. That these factors influence staffing levels is supported by empirical evidence demonstrating that for-profit NHs are generally found to have lower nursing staff levels than not-for-profit NHs (Skinner et al., 2025; White et al., 2023).

## Funding for nursing home care

While NHs rely on a range of revenue sources, Medicaid covers the cost of care for two-thirds of NH residents (National Center for Health Statistics, 2022), making it the largest payer of NH care nationally; however, Medicaid rates do not always meet the reported cost of resident care. Analyses from both the Assistant Secretary for Planning and Evaluation (ASPE) and the Medicaid and CHIP Payment Access Commission (MACPAC) found that Medicaid payment rates covered an average of 83% and 86%, respectively, of the per diem cost of caring for Medicaid residents in NHs in 2019 (Bowblis et al., 2024a; MACPAC, 2023). The ASPE study also documented that, for the average facility, the overall cost of caring for all residents was roughly equal to the total revenue received from all sources. This suggests that NHs are using revenue from non-Medicaid sources (e.g., Medicare, private pay) to supplement the cost of caring for Medicaid residents.

Despite Medicaid being a primary funding source for NHs, and the potentially adverse implications of Medicaid funding shortfalls for staffing and quality, federal statutes do not require that state Medicaid programs cover the full cost of care, instead mandating only that funding levels be “consistent with efficiency, economy, and quality of care and sufficient to enlist enough providers so that the services are available to beneficiaries at least to the extent that those services are available to the general population” (42 CFR § 447.204). Accordingly, substantial variation exists across state Medicaid payment policies and payment levels (Bowblis et al., 2024a; MACPAC, 2019, 2023). For example, states use a combination of retrospective facility-specific cost-based, prospective facility-independent price-based, hybrid, or other rate-setting methodologies carried out at various intervals and incorporate a variety of other factors to settle on a final Medicaid payment amount, such as acuity-based case-mix adjustments, inflation factors, and incentive payments (MACPAC, 2019).

Although states design these payment systems to achieve specific objectives, including quality improvement (Miller et al., 2009), limited research examines the association between Medicaid payments and staffing levels; none has been conducted at the facility level nationally. Nursing staff represents the single largest operational cost in NH care; in fact, NHs spent almost 34% of total revenues on nursing staff in 2019 (Bowblis et al., 2023). Additionally, research indicates that NHs with a larger share of Medicaid residents (also called Medicaid payer mix) spend a higher proportion of revenue on nursing staff, while also having lower overall nursing staff levels; this research also documents that for-profit NHs tend have lower nursing staff levels (Bowblis & Brunt, 2025; Bowblis et al., 2023; Harrington et al., 2007). A recent rule from CMS sought to impose a federal minimum for nursing staff levels beginning in 2026 (Centers for Medicare & Medicaid Services [CMS], 2024), though it is noteworthy that only 20% of NHs were anticipated to meet the mandated minimums (Grabowski & Bowblis, 2023). Further, NHs with a higher Medicaid payer mix were predicted to be even less likely to be able to meet the pending requirements (Elliot et al., 2023). This mandate was vacated by two federal courts, and the effort to establish a federal staffing minimum was subsequently abandoned by CMS, citing multiple reasons, including the moratorium on implementation of the minimum staffing provisions until 2035, concerns from rural and tribal communities, and interpretation of the relevant statutes (CMS, 2025).

Though other factors may impact precisely how increased Medicaid rates affect staffing (e.g., competing financial pressures, state regulatory factors), it is important to better understand the relationship between NHs’ primary source of payment - Medicaid - and nursing staff levels. Adding further complexity to the NH funding landscape, recent industry trends indicate that NH revenues are growing more slowly than costs (Brunt et al., 2024), potentially compromising efforts to sustain or increase NH staffing levels and maintain or improve quality.

Consequently, the purpose of this study was to assess the relationship between Medicaid payment rates and nursing staff levels in NHs across different nursing staff types and across ownership types. By drawing on more recent and potentially more accurate staffing data and recently collected national facility-level data on Medicaid reimbursement, this study serves to fill several gaps in the literature. Most previous studies of the relationship between Medicaid payment rates and staffing use staffing levels obtained from cost reports or annual recertification surveys; this study makes use of the Payroll-Based Journal (PBJ) data to determine staffing levels, which is thought to be an improvement over older ways CMS collected staffing-level data. Additionally, most of the past work examining the impact of Medicaid payment rates on a national basis used statewide average Medicaid payment rates and not facility-specific rates, potentially masking the true relationship between Medicaid payment rates and staffing levels (Cohen & Spector, 1996; Feng et al., 2008; Grabowski, 2004; Harrington et al., 2007; Li et al., 2015). A handful of studies have examined facility-specific rates, but these are limited to a single state and may not be generalizable to other states (Bowblis & Applebaum, 2017; Hackman, 2019; Hyer et al., 2013; Mukamel et al., 2012). Some studies have examined incentive payments embedded in Medicaid payment rates, but these too focus on statewide rather than facility-level incentives (Feng et al., 2010; Konetzka et al., 2018). With few exceptions (Bowblis & Applebaum, 2017; Hackman, 2019), most studies examining the relationship between Medicaid payments or incentives used data more than 15 years old. Moreover, no study to date has explored how and to what extent the association between Medicaid payments and nursing staff levels varies across not-for-profit, for-profit, and government-owned NHs.

It was hypothesized that higher Medicaid payment rates provide NHs with more financial resources, allowing for investment in higher nursing staff levels. It was also hypothesized that the association between Medicaid payment rates and nursing staff levels would vary by ownership type, given that not-for-profit NHs tend to staff at higher levels than for-profit NHs.

## Method

### Data and study sample

The national facility-level dataset used in this study combines statutory Medicaid payment rates collected directly from the states; staffing information collected from the PBJ; and facility and resident characteristics from the Minimum Data Set (MDS), Certification and Survey Provider Enhanced Reporting (CASPER) system, and Medicare Cost Reports.

The unit of analysis is an individual NH’s unique fiscal year as defined by the Medicare Cost Report reporting period. The Medicaid payment rate, staffing data from PBJ, and all variables were constructed so that the data reflect the reporting period most closely aligning with the (full year) Medicare Cost Report for each NH that ended before September 2019. This is the period immediately before the Payment Driven Payment Model was implemented for Medicare. Therefore, the analytic sample reflects NHs with fiscal year-end dates that run from July 2018 to June 2019, with the majority (73.8%) having a fiscal year that runs from January 1 to December 31, 2018.

Medicaid payment rates were obtained from 45 states; usable data could not be obtained from five states (Arizona, Michigan, New Mexico, South Dakota, and Tennessee). Data for these 45 states account for 93% (13,285) of freestanding NHs in the United States. Of these, Medicaid payment rates were successfully matched to facility identifiers in the Medicare Cost Reports for 92% of NHs. NHs were excluded if they reported Medicaid payment rates or estimated Medicaid costs outside the 1% tails, including all NHs in Alaska, reported financials that included non-NH components (e.g., deriving from associated home health settings), and did not report daily staffing levels for all days in the reporting period of the Medicare Cost Report. Sensitivity analyses found that including NHs with other services had little impact on the findings. Although a small proportion of NHs (<1%) do not report staffing hours for every day, the exclusion of these observations is unlikely to impact the findings. MDS assessments were utilized to construct long-stay resident characteristics, and two NHs without MDS long-stay assessments were also excluded. The final analytic sample used a NH as an observation and contained data on 9,510 NHs across 44 states.

### Medicaid payment rate

The primary predictor used for this analysis was the statutory per diem Medicaid payment rate at the facility level. This statutory rate is the per diem amount state law dictates that a NH should receive per day for providing care to a Medicaid resident, regardless of whether the funds are paid by residents or the state. The Medicaid payment rate was standardized across states to facilitate comparisons across states. More details on how these rates were calculated and standardized are available in Bowblis et al. (2024a). For analytic purposes, each NH’s Medicaid payment rates were categorized into the following groups: ≤$175, $176–$200, $201–$225, $226–$250, $251–$275, $276–$300, and $300 or more. A categorical specification utilizing bins of $25 was chosen to support interpretability in the policy context and to account for potential non-linear relationships between Medicaid payment rates and staffing. The specific categories used were selected to ensure that the entire distribution of Medicaid payment rates was represented with a sufficient number of observations in each category.

### Nursing staff levels

Nursing staff levels were drawn from the PBJ data and were measured in HPRD. In 2017, CMS made the PBJ data available, which, compared with how staffing data was collected in the past, is thought to provide more accurate information on nursing staff levels. Nursing staff levels were calculated for each day by dividing hours worked by the estimated census, then aggregating to the average staffing level over the reporting period of the cost report for each NH. Separate nursing staff levels were calculated for RNs, LPNs, nurse aides, and total nursing staff (the sum of RNs, LPNs, and nurse aides). In defining which job codes to include for each type of nursing staff, CMS’ definitions for the Care Compare website were utilized (CMS, 2026).

### Ownership type

Ownership type derived from the Medicare Cost Reports. Facilities were identified as not-for-profit, for-profit, or government-operated.

### Covariates

Facility and resident characteristics were controlled for in the regression analyses. Facility characteristics were obtained from the Medicare Cost Reports, including the number of beds, occupancy rate, Medicare payer-mix, Medicaid payer-mix, chain affiliation, and rural location. Facility characteristics were also obtained from CASPER, including whether the facility was part of a Continuing Care Retirement Community (CCRC) and whether it had a Memory Care Unit or other special care unit. Resident characteristics were derived from the MDS and aggregated to the facility level for long-stay residents, including age; female sex; black, indigenous, and other people of color (BIPOC); dementia; RUG-IV Activities of Daily Living (ADL) Score (CMS, 2017); and severe mental illness (SMI; Bipolar disorder, Schizophrenia, or other psychotic disorder). Average long-stay resident characteristics were derived by obtaining the first MDS assessment for each unique long-stay resident (i.e., length of stay >100 days) in the fiscal year in which Medicaid payment rates are collected, then aggregating to the facility level.

### Analysis

To understand the association between Medicaid payment rates and nursing staff levels, three sets of analyses were performed. First, descriptive statistics were calculated for Medicaid payment rates and covariates. These descriptive statistics included examining the average HPRD for RNs, LPNs, nurse aides, and total nursing staff across all facilities and by ownership type (not-for-profit, for-profit, government). Second, the analysis assessed the overall relationship between Medicaid payment rates and nursing staff levels. This was first done by examining bivariate associations and then moving to multivariate regression analysis. Each regression had a measure of nursing staff level as the dependent variable, with the key explanatory variable being the Medicaid payment rate category. These regressions captured differences in ownership by including indicator variables for each ownership type and controlled for facility and resident characteristics and state-fixed effects. Due to the cross-sectional nature of the analysis, state fixed effects account for potentially unobserved and time-invariant differences across states, such as cost-of-living differences or minimum staffing regulations. Finally, using similar bivariate and multivariate regression models, subgroup analyses were performed that restricted the sample to NHs of only a specific ownership type. Because these analyses were limited to one type of ownership, the ownership type variable was not included in these regression models. All regressions reported robust standard errors that adjusted for clustering of facility-level observations within states to account for how staffing levels can be influenced by statewide NH policies. The study protocol was reviewed by the University of Massachusetts Institutional Review Board and determined to be exempt, as it did not constitute human subjects research under 45 CFR §46.102

## Results

### Summary statistics

Descriptive statistics for Medicaid payment, facility characteristics, and long-stay resident characteristics are reported in Table S1. Most NHs had Medicaid per diem payment rates of ≤$175 (30.1%), $176–$200 (25.1%), and $201–$225 (23.6%). Only a little more than one-fifth (21.0%) had payment rates above $225 per day. For-profit NHs constituted the majority of the sample (74.0%), followed by not-for-profit (18.0%) and government NHs (8.0%). Most NHs (54.1%) were affiliated with a chain; few were part of a CCRC (6.2%). The average number of beds was nearly 115, with an average occupancy rate of 80.7%. At 15.6% and 5.8%, respectively, memory care units were more common than other special care units. Average payer mix was 58.1% for Medicaid and 12.0% for Medicare. Rural NHs accounted for 26.7% of the sample. For the average NH, long-stay residents averaged 78.2 years of age; 64.6% were female, and 22.2% were BIPOC. The percentage of residents with dementia and serious mental illness was 49.7% and 18.3%, respectively. The average RUG-IV ADL score was 7.658.

Table 1 reports nursing staff levels for all NHs and by ownership type. Nursing staff levels averaged 0.58, 0.87, and 2.26 HPRD for RNs, LPNs, and nurse aides, respectively; total nursing staff HPRD was 3.70. Not-for-profit NHs exhibited the highest total nursing staff levels (4.03 HPRD), followed by government NHs (3.69) and for-profit NHs (3.62). Whereas average RN and nurse aide HPRD were highest in not-for-profits, LPN HPRD in nonprofit NHs (0.81) were lower than for-profit (0.88) and government NHs (0.90).

**Table 1:** Nursing staff levels (in HPRD) for all facilities and by ownership type.

Nursing staff level	All facilities (*N *= 9,510)	For-profit (*n *= 7,034)	Not-for-profit (*n *= 1,718)	Government (*n *= 758)
**Registered nurses**	0.58	0.54	0.73	0.54
**Licensed practical nurses**	0.87	0.88	0.81	0.90
**Nurse aides**	2.26	2.20	2.49	2.26
**Total nursing staff**	3.70	3.62	4.03	3.69

*Note.* HPRD = hours per resident day.

### Medicaid payment rates and staffing levels

Table 2 shows the bivariate results comparing average nursing staff levels by Medicaid payment rate. Results indicate that staffing levels rose progressively as Medicaid payment rates increased for RNs, nurse aides, and total nursing staff, but not for LPNs. Thus, average RN, nurse aide, and total nursing staff rose from 0.46, 2.14, and 3.43 HPRD at Medicaid payment rates of ≤$175 per day, respectively, to 0.81, 2.63, and 4.19 HPRD at $300+ per day. Average LPN HPRDs were lowest at the highest Medicaid payment rates (0.79 and 0.76, respectively, for $276–$300 and $300+ vs 0.84 or higher for the five lowest Medicaid payment rate categories).

**Table 2:** Average nursing staff levels (in HPRD) by Medicaid payment rate.

Medicaid payment rate	RNs	LPNs	Nurse aides	Total nursing staff
**≤$175**	0.46	0.84	2.14	3.43
**$176–$200**	0.55	0.88	2.20	3.63
**$201–$225**	0.62	0.89	2.29	3.81
**$226–$250**	0.67	0.89	2.41	3.98
**$251–$275**	0.75	0.87	2.45	4.07
**$276–$300**	0.77	0.79	2.49	4.05
**$300+**	0.81	0.76	2.63	4.19

*Note. N *= 9,510. HPRD = hours per resident day; LPN = licensed practical nurse; RN = registered nurse.

Table 3 reports the regression results examining the association between Medicaid payment rates and staffing levels after controlling for ownership, facility/resident characteristics, and state fixed effects. With the exception of LPNs, higher Medicaid payment rates were associated with a monotonic increase in staffing levels for RNs, nurse aides, and total nursing staff. For example, relative to the lowest Medicaid payment rate (≤ $175 per day), NHs with the highest Medicaid payment rates ($300+) had higher RN, nurse, and total nursing staff levels of 0.25, 0.31, and 0.52 HPRD, respectively. For LPNs, compared to the lowest Medicaid payment rate, moderately higher rates (≤ $175 vs $176–$250) were associated with higher LPN staffing levels but were not statistically significant once rates exceeded $250 per day.

**Table 3:** Regression results: nursing staff levels (in HPRD) by Medicaid payment rate controlling for other characteristics.

Medicaid payment rate	RNs	LPNs	Nurse aides	Total nursing staff
**≤$175**	Ref	Ref	Ref	Ref
**$176–$200**	0.044***	0.027**	0.048	0.119**
	**(0.014)**	(0.013)	(0.045)	(0.051)
**$201–$225**	0.075***	0.043**	0.125**	0.243***
	**(0.016)**	(0.018)	(0.050)	(0.063)
**$226–$250**	0.106***	0.059**	0.150***	0.315***
	**(0.023)**	(0.027)	(0.051)	(0.068)
**$251–$275**	0.174***	0.047	0.187***	0.408***
	**(0.027)**	(0.050)	(0.058)	(0.095)
**$276–$300**	0.213***	−0.010	0.232***	0.434***
	**(0.054)**	(0.076)	(0.076)	(0.151)
**$300+**	0.247***	−0.034	0.311***	0.524***
	**(0.053)**	(0.084)	(0.088)	(0.183)
***R* ^2^**	.501	.341	.368	.376

*Note. N *= 9,510. HPRD = hours per resident day; RN = registered nurse; LPN = licensed practical nurse. The effect of Medicaid payment rates was estimated using linear regression models that controlled for Medicaid payment rate categories, ownership type, all facility and resident characteristics listed in Table 1, and state fixed effects. All standard errors were adjusted for the clustering of nursing homes within states.

*** *p *< .01.

** *p *< .05.

* *p *< .1.

### Medicaid reimbursement and total nursing staff levels by ownership type

Because staffing levels have been historically different by ownership type, Table 4 reports the average total nursing staff level by Medicaid payment rate across the three ownership types. Regardless of ownership type, higher Medicaid payment rates were associated with higher total nursing staff levels. For-profit NHs exhibited the lowest staffing levels for each Medicaid payment rate category. Not-for-profit NHs exhibited the highest total nursing staff levels at the three lowest Medicaid payment rate categories (≤$175, $176–$200, $200–$225) and at the highest category ($300+), with government NHs being equal to or higher than not-for-profits for Medicaid payment categories between $226 and $275.

**Table 4:** Average total nursing staff levels (in HPRD) by Medicaid payment rate for nursing homes of each ownership type.

Medicaid payment rate	For profits (*n *= 7,034)	Not-for-profits (*n *= 1,718)	Government (*n *= 758)
**≤$175**	3.35	3.79***	3.52***
**$176–$200**	3.55	3.93***	3.65**
**$201–$225**	3.75	4.07***	3.83**
**$226–$250**	3.92	4.16***	4.17**
**$251–$275**	3.93	4.38***	4.38***
**$276–$300**	3.82	4.54***	4.62***
**$300+**	4.09	4.56***	4.48

*Note. N *= 9,510. HPRD = hours per resident day. Statistical tests (*t-*tests) compared whether not-for-profit and government nursing homes had statistically different staffing levels within each Medicaid payment rate category.

*** *p *< .01.

** *p *< .05.

* *p *< .1.

To assess whether this relationship holds after controlling for differences in NHs across ownership types, Table 5 reports the subgroup regression results. The results confirmed that as Medicaid payment rates increased, regardless of ownership type, total nursing staff levels increased, though the effect was attenuated at the highest payment category among government NHs. Generally, not-for-profit and government NHs seemed to be more responsive to Medicaid payment rate increases than for-profit NHs. For example, increasing Medicaid payment rates from the ≤$175 category to the $251–$275 category was associated with a 0.58 HPRD increase in total nursing staff levels for both not-for-profit and government NHs, but only a 0.35 HPRD increase among for-profits. This pattern existed for all payment categories except the second lowest (i.e., $176–$200).

**Table 5:** Regression results: total nursing staff levels (in HPRD) by Medicaid payment rate controlling for other characteristics for nursing homes of each ownership type.

Medicaid payment rate	For profits (*n *= 7,034)	Not-for-profits (*n *= 1,718)	Government (*n *= 758)
**≤$175**	Ref	Ref	Ref
**$176–$200**	0.119**	0.104*	0.210***
	**(0.051)**	(0.056)	(0.055)
**$201–$225**	0.230***	0.269***	0.433***
	**(0.071)**	(0.084)	**(0.070)**
**$226–$250**	0.302***	0.384***	0.633***
	**(0.070)**	(0.090)	**(0.153)**
**$251–$275**	0.351***	0.575***	0.585**
	**(0.082)**	*(0.157)*	(0.219)
**$276–$300**	0.344**	0.639***	0.798**
	**(0.132)**	**(0.182)**	(0.377)
**$300+**	0.437***	0.777***	−*0.150*
	**(0.152)**	*(0.258)*	*(0.418)*
***R* ^2^**	.455	.402	.160

*Note.* HPRD = hours per resident day. The effect of Medicaid payment rates on total nursing staff level was estimated using linear regression models that controlled for Medicaid payment rate categories, all facility and resident characteristics listed in Table 1, and state fixed effects. Separate regressions were estimated for nursing homes with each ownership type. All standard errors adjusted for the clustering of nursing homes within states. Italic and bold coefficient estimates indicate that the effect for a not-for-profit or government nursing home, relative to a for-profit nursing home, is statistically different at the 10% and 5% levels, respectively.

*** *p *< .01.

** *p *< .05.

* *p *< .1.

Additional analyses (not shown) were conducted to examine the relationship between Medicaid payment rates and staffing levels for RNs, LPNs, and nurse aides by ownership type. Findings for RNs and nurse aides match the total nursing staff findings; however, no statistically different relationships were evident between Medicaid payment rates and LPN staffing levels for any ownership type.

## Discussion and conclusion

Utilizing national data, this study provides evidence that Medicaid payment rates are strongly associated with NH staffing levels, underscoring the key role that state Medicaid payment policies play in influencing the quality of care. Across all NHs, higher Medicaid payment rates were consistently associated with higher staffing levels for RNs, nurse aides, and total nursing staff. Moreover, these associations were consistent among for-profit, not-for-profit, and government-owned NHs, though their responsiveness to higher payment rates varied. In contrast, higher Medicaid payment was not consistently associated with LPN staffing levels, either overall or by ownership type.

This research serves as an important addition to the literature that has previously demonstrated that Medicaid payment rates and incentives can bolster NH staffing levels (Bowblis & Applebaum, 2017; Cohen & Spector, 1996; Feng et al., 2010; Grabowski, 2004; Hackman, 2019; Harrington et al., 2007; Hyer et al., 2013; Konetzka et al., 2018; Li et al., 2015; Mukamel et al., 2012). Moreover, it addresses a gap in the literature by using a nationwide, facility-level dataset, in contrast to prior literature’s reliance on statewide averages or facility-level data for a single state. Because the dataset used in the present study collects Medicaid payment data at the facility level across 44 states, it more convincingly demonstrates the association between Medicaid payment rates and nursing staff levels in NHs, when adjusting for facility and resident characteristics. Not only do the findings indicate that higher Medicaid payment was correlated with higher total nursing staff levels, but also that higher payments bolster staffing levels for nurse aides and RNs, the two types of nursing staff that were the focus of CMS’ attempt to implement a federal minimum staffing standard in 2024 (CMS, 2024). The results for RNs are notable given evidence linking RN staffing to quality outcomes (Armijo-Olivo et al., 2020; Brunt, 2023; Clemens et al., 2021; Dellefield et al., 2015; Millar et al., 2024).

For LPNs, a truncated effect was found, with Medicaid payment rates only up to $250 per day associated with higher LPN staffing levels; there was no statistically significant difference in LPN staffing at Medicaid payment rates above $250 per day. The limited association between Medicaid payment and LPN staffing is consistent with prior research that found Medicaid payments are generally associated with RN and nurse aide staffing (Bowblis & Applebaum, 2017; Cohen & Spector, 1996; Li et al., 2015; Mukamel et al., 2012). More importantly, when combined with the results for RNs, the LPN findings suggest that, with higher Medicaid payments, NHs may turn to RNs over LPNs when increasing their licensed nursing staff levels. This pattern may emerge if low Medicaid rates limit a NH’s financial resources, leading NHs to hire lower-paid LPNs over higher-paid RNs to ensure they have sufficient licensed staff to meet the needs of residents or regulatory requirements. Conversely, once Medicaid payment rates hit a critical threshold, NHs may perceive more flexibility in staffing decisions and choose to hire RNs over LPNs due to their greater training, scope of practice, and stricter licensure requirements.

Within the context of ownership type, this study found that not-for-profit NHs staff at higher levels than for-profit NHs, consistent with previous work (White et al., 2023). Some observers have argued that some for-profit NH operators prioritize profits over investing in staff and quality (Harrington et al., 2015). Yet, regardless of ownership type, our results found that higher Medicaid payment rates are associated with higher RN, nurse aide, and total nursing staff levels. This suggests that when Medicaid payment rates are higher, all NHs, including for-profit NHs, increase staffing levels. This is an especially important finding given for-profit NHs’ disproportionate reliance on Medicaid as a revenue source.

Despite similarities, there are notable differences in the responsiveness to increases in Medicaid payment rates across ownership types. The association between Medicaid reimbursement and total nurse staffing is generally comparable across for-profit and not-for-profit NHs at lower average per diem payments (≤$250). However, the association between Medicaid payment and staffing levels among for-profits is not as substantial as among not-for-profits at the highest reimbursement levels (>$250). While our analysis cannot tease out why this occurs, there are many potential reasons. For example, once Medicaid payment rates hit a critical level, not-for-profit NHs may continue to invest in nursing staff, while for-profit NHs may invest these additional funds in other areas that enhance quality of care and life, such as therapy staff, activities, food quality, and other amenities. Alternatively, higher payment rates might be associated with greater profits, which for-profit NHs either invest in other properties or return to owners, options that are not available to not-for-profits due to the non-distribution constraint. In this scenario, increases in Medicaid payment rates that are greater than the cost of inflation should be tied to transparent investments by NHs in nursing staff, ancillary services staff, or other quality improvement efforts. Finally, less than one in ten NHs in the study sample (9.9%) had an average Medicaid per diem rate over $250; consequently, the results could be due to small sample sizes. Further investigation is needed to understand what is driving the differences in staffing between not-for-profit and for-profit NHs at higher levels of Medicaid payment.

These findings carry important implications for policymakers. Recent research confirming that Medicaid often reimburses below the costs of care highlights a structural funding gap that could constrain staffing capacity (Bowblis et al., 2024a; MACPAC, 2023). Because staffing is one of the single largest expenses for NHs (Bowblis et al., 2023), Medicaid’s failure to cover full costs may exacerbate staffing shortages, turnover, and reliance on agency staff that compromise quality of care and contribute to adverse resident outcomes (Bowblis et al., 2024b). As labor costs rise, study results suggest that NHs may not be able to meet existing or, in some states, newly enacted higher staffing minimum requirements unless provided with additional resources through higher Medicaid payment, particularly in facilities with a high Medicaid payer-mix (Bowblis & Brunt, 2025; Elliot et al., 2023). These increases could entail broader adjustments in Medicaid base payment rates or reimbursement enhancements directed specifically at staffing. Evidence from state programs employing wage pass-through and pay-for-performance incentives suggests that targeted Medicaid policies can bolster staffing capacity, though the effect could vary by ownership type due to different baseline staffing levels, state-level policy design, and organizational behaviors (Feng et al., 2010; Foster & Lee, 2015; Konetzka et al., 2018; Miller et al., 2012).

This study has several limitations. First, the analysis was based on data from 2019, and there have been several changes in the NH landscape since the end of the public health emergency brought on by the COVID-19 pandemic. Several states have raised their Medicaid payment rates (KFF, 2023), wages and the cost of borrowing have increased (Bowblis et al., 2024a), and Medicare implemented the Patient Driven Payment Model (CMS, 2018). Second, Medicaid payment rates may exclude certain revenue sources, such as payments made directly by Medicaid residents and lump-sum supplemental payments. Nonetheless, prior analyses indicate that these omissions did not significantly distort the calculated payment levels. Third, because the analysis used state-fixed effects to account for cross-state variation, the study could not determine if the mechanisms used by states to determine Medicaid payment rates (e.g., price-based vs cost-based systems; rebasing frequency) could impact how much a facility is actually paid and thus how it may impact staffing. The use of state fixed effects also cannot control for within-state variability, constituting a potential source of bias. Both of these may be useful areas for future research. Fourth, Payroll-Based Journal data relies on self-report to document NH staffing levels. This data is thus vulnerable to the accuracy issues associated with self-reported data. Yet, the Payroll-Based Journal data is subject to audits and has been used in numerous other studies (Brunt et al., 2024; Kang et al., 2025; Mukamel et al., 2024) and is widely regarded as the best source of data available on nursing staff levels in NHs (Williams et al., 2019). Fifth, several decisions were made regarding sample inclusion to ensure that the study analyzed the most homogeneous group of NHs. If the excluded NHs were significantly different from the study sample, this could affect the generalizability of the study findings. Sixth, cross-sectional data were used in this analysis to examine the association between Medicaid payment levels and nursing staff levels. Because Medicaid payment rates are determined using an NH’s historical operating cost or a price-based system where rates are similar across all NHs with similar acuity residents, endogeneity caused by current staffing levels determining current Medicaid payment rates is not a concern. Further, the cross-sectional design meant causality could not be determined and also precluded the investigation of intermediary pathways operating between Medicaid payment and staffing levels; this could be an area for future research using longitudinal data. Last, although the results found lower nursing staff levels among NHs with lower Medicaid payment levels and long-stay Medicaid residents have lower acuity (i.e., staffing needs) than post-acute care patients per CMS billing systems, this study’s analysis was not designed to determine if these lower nursing staff levels were associated with being adequately staffed.

In conclusion, this study supports a positive association between Medicaid payment rates and RN, nurse aide, and total nursing staff levels; conversely, Medicaid payment rates were not consistently associated with LPN staffing. While not-for-profit NHs generally staff at higher levels, the findings indicate that increases in Medicaid per diem payment may enhance staffing capacity among not-for-profit, for-profit, and government NHs alike, supporting the likelihood of a broad and consistent influence of reimbursement policy on staffing. Given the importance of staffing for quality of care in the NH setting, these findings demonstrate that Medicaid is an important policy lever for furthering resident well-being. Higher payment could be particularly important for improving the quality of care in NHs that rely heavily on Medicaid, including NHs located in rural communities and those serving black and indigenous people of color and other disadvantaged populations (Bowblis et al., 2013; Feng et al., 2011), constituting an important potential area for future research.

## Supplementary Material

igag087_Supplementary_Data

## Data Availability

Most data used in this study derive from publicly available data sources. However, the facility-specific data on Medicaid nursing home payments are currently not publicly available since they are part of an ongoing research project. Reasonable requests for access may be considered case-by-case by the corresponding author. This study was not preregistered.
