# Supplementary material for: Medicaid payment rates and nursing home staffing levels across ownership types
Source: Innov Aging. 2026 Jul 22;10(10):igag087. doi: 10.1093/geroni/igag087 (PMC13585398; doi:10.1093/geroni/igag087)
Supplement: Supplementary file 1 [file igag087_supplementary_data.pdf]

Supplemental Materials: Full Regression Tables

Supplemental Table 1. Regression Results: Average Nursing Staff Levels (in HPRD) by Medicaid Payment Rate

VARIABLES	RNs	LPNs	Nurse Aides	Total Nursing Staff
1- ≤-175	Ref	Ref	Ref	Ref
2- 176-200	0.044***	0.027**	0.048	0.119**
	(0.014)	(0.013)	(0.045)	(0.051)
3- 201-225	0.075***	0.043**	0.125**	0.243***
	(0.016)	(0.018)	(0.050)	(0.063)
4- 226-250	0.106***	0.059**	0.150***	0.315***
	(0.023)	(0.027)	(0.051)	(0.068)
5- 251-275	0.174***	0.047	0.187***	0.408***
	(0.027)	(0.050)	(0.058)	(0.095)
6- 276-300	0.213***	-0.010	0.232***	0.434***
	(0.054)	(0.076)	(0.076)	(0.151)
7- 300+	0.247***	-0.034	0.311***	0.524***
	(0.053)	(0.084)	(0.088)	(0.183)
For-profit	-0.087***	-0.002	-0.169***	-0.258***
	(0.016)	(0.015)	(0.033)	(0.046)
Government	-0.027	0.032*	-0.053	-0.049
	(0.024)	(0.017)	(0.075)	(0.096)
Number of Beds	-0.000***	0.000	-0.000	-0.000***
	(0.000)	(0.000)	(0.000)	(0.000)
Chain	0.025***	-0.005	-0.111***	-0.090***
	(0.006)	(0.009)	(0.024)	(0.029)
Part of CCRC	0.031***	0.068***	0.119***	0.218***
	(0.011)	(0.016)	(0.038)	(0.047)
Occupancy Rate	-0.002***	-0.002***	0.001	-0.003***
	(0.000)	(0.000)	(0.001)	(0.001)
% Medicaid payer-mix	-0.001***	-0.001***	-0.001***	-0.003***
	(0.000)	(0.000)	(0.000)	(0.001)
% Medicare payer-mix	0.006***	0.003***	0.000	0.009***
	(0.001)	(0.001)	(0.001)	(0.002)
Memory Care Unit	-0.021***	0.019**	0.056***	0.054***
	(0.006)	(0.008)	(0.017)	(0.019)
Other Special Care Unit	0.073***	0.091**	0.047**	0.212***
	(0.015)	(0.034)	(0.021)	(0.047)
Rural Location	-0.029*	-0.025**	0.031	-0.024
	(0.015)	(0.010)	(0.020)	(0.027)
Average Resident Age	0.004**	-0.007***	0.011***	0.007**
	(0.001)	(0.003)	(0.002)	(0.003)

% Female	-0.001**	0.000	-0.001	-0.002
	(0.000)	(0.000)	(0.001)	(0.001)
% BIPOC	-0.000	-0.000	-0.000	-0.001*
	(0.000)	(0.000)	(0.000)	(0.001)
Average RUG-IV ADL Score	0.015***	0.016**	0.030***	0.060***
	(0.003)	(0.008)	(0.005)	(0.007)
% Dementia	-0.001***	-0.001*	0.001**	-0.000
	(0.000)	(0.000)	(0.001)	(0.001)
% SMI	-0.001	-0.002***	-0.001	-0.004**
	(0.000)	(0.000)	(0.001)	(0.001)
Constant	0.492***	1.506***	1.303***	3.301***
	(0.106)	(0.183)	(0.196)	(0.278)
Observations	9,510	9,510	9,510	9,510
R-squared	0.501	0.341	0.368	0.376

*** p<0.01, ** p<0.05, * p<0.1

Note: All standard errors adjusted for the clustering of observations within states. HPRD=Hours per resident day; RN=Registered nurse; LPN=Licensed practical nurse; BIPOC=Black, Indigenous or People of Color; CCRC=Continuing Care Retirement Community; HPRD=Hours Per Resident Day RUG-IV ADL=Resource Utilization Groups-IV Activity Daily of Living; SMI=Diagnoses of Bipolar, Schizophrenia, other psychotic disorder

Supplemental Table 2. Regression Results: Total Nursing Staff Levels (in HPRD) by Medicaid Payment Rate for All NHs and NHs of Each Ownership Type

VARIABLES	All Facilities	NFPs	FPs	GOV
1- ≤-175	Ref	Ref	Ref	Ref
2- 176-200	0.119**	0.104*	0.119**	0.210***
	(0.051)	(0.056)	(0.051)	(0.055)
3- 201-225	0.243***	0.269***	0.230***	0.433***
	(0.063)	(0.084)	(0.071)	(0.070)
4- 226-250	0.315***	0.384***	0.302***	0.633***
	(0.068)	(0.090)	(0.070)	(0.153)
5- 251-275	0.408***	0.575***	0.351***	0.585**
	(0.095)	(0.157)	(0.082)	(0.219)
6- 276-300	0.434***	0.639***	0.344**	0.798**
	(0.151)	(0.182)	(0.132)	(0.377)
7- 300+	0.524***	0.777***	0.437***	-0.150
	(0.183)	(0.258)	(0.152)	(0.418)
For-profit	-0.258***			
	(0.046)			
Government	-0.049			
	(0.096)			
Number of Beds	-0.000***	-0.001***	-0.000**	0.001
	(0.000)	(0.000)	(0.000)	(0.002)
Chain	-0.090***	-0.192***	-0.065**	-0.128
	(0.029)	(0.034)	(0.031)	(0.082)
Part of CCRC	0.218***	0.199***	0.161***	0.048
	(0.047)	(0.065)	(0.050)	(0.140)
Occupancy Rate	-0.003***	0.000	-0.004***	-0.005**
	(0.001)	(0.002)	(0.001)	(0.002)
% Medicaid payer-mix	-0.003***	-0.004***	-0.002***	-0.006***
	(0.001)	(0.001)	(0.001)	(0.002)
% Medicare payer-mix	0.009***	0.010***	0.010***	-0.003
	(0.002)	(0.003)	(0.002)	(0.007)
Memory Care Unit	0.054***	0.097**	0.036	-0.008
	(0.019)	(0.039)	(0.023)	(0.060)
Other Special Care Unit	0.212***	0.189**	0.214***	0.024
	(0.047)	(0.090)	(0.046)	(0.108)
Rural Location	-0.024	-0.111***	0.003	-0.049
	(0.027)	(0.034)	(0.029)	(0.042)
Average Resident Age	0.007**	0.004	0.002	0.011
	(0.003)	(0.007)	(0.003)	(0.009)
% Female	-0.002	0.000	0.000	-0.006**
	(0.001)	(0.003)	(0.001)	(0.003)
% BIPOC	-0.001*	-0.002	-0.001	0.000

	(0.001)	(0.001)	(0.000)	(0.002)
Average RUG-IV ADL Score	0.060***	0.044***	0.068***	0.056**
	(0.007)	(0.013)	(0.007)	(0.022)
% Dementia	-0.000	-0.001	0.000	-0.002
	(0.001)	(0.001)	(0.001)	(0.003)
% SMI	-0.004**	-0.007***	-0.003**	-0.000
	(0.001)	(0.002)	(0.001)	(0.002)
Constant	3.301***	3.531***	3.241***	3.452***
	(0.278)	(0.508)	(0.284)	(0.733)
Observations	9,510	1,718	7,034	758
R-squared	0.376	0.402	0.455	0.160

*** p<0.01, ** p<0.05, * p<0.1

Note: All standard errors adjusted for the clustering of observations within states. HPRD=Hours per resident day; NFP=not-for-profit; FP=for-profit, GOV=government; BIPOC=Black, Indigenous or People of Color; CCRC=Continuing Care Retirement Community; HPRD=Hours Per Resident Day RUG-IV ADL=Resource Utilization Groups-IV Activity Daily of Living; SMI=Diagnoses of Bipolar, Schizophrenia, other psychotic disorder

Supplemental Table 3. Regression Results: RN, LPN, and Nurse Aide Staffing Levels (in HPRD) by Medicaid Payment Rate Controlling for Other Characteristics for NHs of Each Ownership Type

	**Registered Nurses**				**Licensed Practical Nurses**				**Nurse Aides**		
**Medicaid Payment Rate ($)**	NFPs	FPs	GOV		NFPs	FPs	GOV		NFPs	FPs	GOV
**1- ≤175**	Ref	Ref	Ref		Ref	Ref	Ref		Ref	Ref	Ref
**2- 176-200**	0.053**	0.047***	0.066**		0.021	0.020	0.015		0.031	0.052	0.128
	(0.025)	(0.017)	(0.028)		(0.027)	(0.014)	(0.047)		(0.053)	(0.045)	(0.079)
**3- 201-225**	0.067**	0.085***	0.093***		0.021	0.040*	0.063		0.181**	0.105*	0.276***
	(0.027)	(0.020)	(0.022)		(0.035)	(0.023)	(0.046)		(0.071)	(0.056)	(0.082)
**4- 226-250**	0.062	0.126***	0.152**		0.044	0.053*	0.132		0.278***	0.123**	0.349***
	(0.039)	(0.025)	(0.064)		(0.052)	(0.028)	(0.114)		(0.072)	(0.052)	(0.110)
**5- 251-275**	0.155**	0.176***	0.243***		0.087	0.024	0.125		0.333***	0.150**	0.217
	(0.058)	(0.032)	(0.069)		(0.102)	(0.042)	(0.125)		(0.084)	(0.057)	(0.156)
**6- 276-300**	0.292***	0.174***	0.168**		0.029	-0.038	0.242		0.318**	0.208***	0.388*
	(0.074)	(0.051)	(0.076)		(0.119)	(0.065)	(0.250)		(0.135)	(0.067)	(0.215)
**7- 300+**	0.304***	0.214***	0.004		0.024	-0.055	-0.037		0.449***	0.279***	-0.117
	(0.096)	(0.043)	(0.115)		(0.107)	(0.077)	(0.268)		(0.148)	(0.076)	(0.285)

N=9,510

*** $p<0.01$, ** $p<0.05$, * $p<0.1$

Note: HPRD=Hours per resident day; RN=Registered nurse; LPN=Licensed practical nurse; NFP=not-for-profit; FP=for-profit, GOV=government The effect of Medicaid payment rates on total nursing staff level was estimated using linear regression models that controlled for Medicaid payment rate categories, all facility and resident characteristics listed in Table 1, and state fixed effects. Separate regressions were estimated for nursing homes with each ownership type. All standard errors adjusted for the clustering of nursing homes within states.

.
